# Needling Therapy for Myofascial Pain Control

**DOI:** 10.1155/2013/946597

**Published:** 2013-08-26

**Authors:** Chang-Zern Hong

**Affiliations:** ^1^Department of Physical Therapy, Hungkuang University, Sha-Lu, Tai-Chung 433, Taiwan; ^2^Department of Physical Medicine and Rehabilitation, University of California Irvine, Irvine, CA 92818, USA

Needling therapy has been widely used for pain control. “Needling” includes all procedures with penetration of a needle through the skin with injection of medication (injection therapy) or without introduction of any drug (dry needling). In either injection or dry needling, the site of treatment can be at the site of pain (direct needling) or far from the site of pain (remote needling). Needling therapy includes traditional acupuncture, needling with multiple insertions, dry needling with electrical stimulation, superficial needling, and Fu's subcutaneous dry needling. 

Myofascial pain is a regional pain syndrome characterized with the existence of a sensitive spot (myofascial trigger point (MTrP)) in the taut band of skeletal muscle fibers. MTrP can be inactivated by appropriate treatment with physical therapy (including manual therapy) or needling therapy. 

This special issue contains nine papers, of which two are review articles and seven are research articles. The review paper by L.-W. Chou et al. discussed various proposed mechanisms published previously and concluded that multiple mechanisms are probably involved in needling analgesia. Another review paper by J. M. Climent et al. discussed the clinical trials of botulinum toxin injection for treating myofascial pain in the neck and back, and concluded that botulinum toxin could be useful in specific myofascial pain regions, especially for patients with refractory myofascial pain that has not responded to other myofascial injection therapies. Two clinical studies demonstrated the effectiveness of dry needling in treating myofascial pain after total knee arthroplasty (O. Mayoral et al.) and remote pain in the upper trapezius muscle (K.-H. Chen et al.). One animal study by Y.-L. Hsieh et al. showed that dry needling at the myofascial trigger spots (MTrSs, equivalent to human MTrP) of rabbit biceps femoris muscles could modulate various biochemicals associated with pain, inflammation, and hypoxia in a dose-dependent manner. The other animal study by A. Domingo et al. found that repeated dry needling punctures in muscle do not perturb the different stages of muscle regeneration and reinnervation. Another research study by M.-T. Lin et al. suggested that the artificial neural network (ANN) model was more accurate in predicting patient-reported pain scores and had higher overall performance indices. Finally two papers reported their newly developed techniques for myofascial pain control. An animal study by Fu et al. further confirm the effectiveness and the possible mechanism of Fu's remote needling for myofascial pan therapy. The study by Lin et al. demonstrated the effectiveness of Lin's percutaneous soft tissue release (less invasive than the surgical release) for treating chronic recurrent myofascial pain due to lateral epicondylitis. 

Many studies in this issue reported the application of multiple insertion technique for dry needling therapy. In either clinical practice or research studies, it has been suggested that needling with “multiple insertion technique” can usually provide the best and fastest effects for immediate pain control. This technique was originally described by Travell and Bobb for myofascial trigger point (MTrP) injection. During injection, the needle was moved in and out into different directions to elicit painful sensation (to encounter the sensitive loci) in an MTrP region. In this way, MTrP pain can usually be eliminated nearly completely immediately after most of those multiple sensitive loci have been injected (or encountered). Considering the time consuming and the possibility of muscle fiber damage during this slow needle movement, C.-Z. Hong has modified this technique into a “fast-movement procedure” in order to avoid tissue damage from side movement of needle or grab of needle by the elicited local twitch responses (LTRs). Later, this new technique has been recommended by Simons and has been widely used for MTrP injection or dry needling. 

The descending pain inhibitory system is probably involved in the mechanism of immediate pain relief after multiple needle insertion. Either hyperstimulation analgesia for general pain control or disruption of “MTrP” circuit for myofascial pain control is actually via the descending inhibitory system. It has been suggested that eliciting LTRs (feeling similar to “De-Qui” effect) during needling is essential to obtain an immediate and complete pain relief after MTrP needling or MTrP injection. Eliciting an LTR or obtaining “De-Qi” effect indicates that a sensitive locus (sensitized nociceptor) is encountered by the needle tip. It appears that, when a sensitive locus is encountered by the tiny needle tip, “De-Qi” effect can be perceived and an LTR can be elicited, and then the irritability of this sensitive locus can be suppressed immediately. Needling of a key MTrP can also inhibit the irritability of satellite MTrPs due to central desensitization phenomenon. It is important to provide strong stimuli to the sensitive loci as many as possible to obtain optimal hyperstimulation in order to have maximal pain relief; so that the “multiple insertion technique” can provide better effects than just a single site stimulation. 

Based on the reports in this special issue, it is very likely that the above issues have been further clarified. 

*Chang-Zern Hong* *Chang-Zern Hong*

